# Associations between physical activity, socioeconomic and sociodemographic factors, and risk of sarcopenia in older adults in Northeast Brazil

**DOI:** 10.1186/s12889-025-25158-x

**Published:** 2025-11-10

**Authors:** Matheus Guerra, Danilo Garcia, Thiago Medeiros da Costa Daniele, Daniel Berglind, João Guerra, Brenda Pinheiro Evangelista, Francisco Gerlai Lima Oliveira, Marília Braga Marques, Rachel Gabriel Bastos Barbosa, Janaína Fonseca Victor Coutinho

**Affiliations:** 1 Department of Global Health, Karolinska Institute, Stockholm, Sweden; 2 Department of Social Studies, Promotion of Health and Innovation for Well-Being (PHI-WELL), University of Stavanger, Stavanger, Norway; 3 Department of Social Studies, University of Stavanger, Stavanger, Norway; 4 Lab for Biopsychosocial Personality Research (BPS-PR), International Network for Well- Being, Stavanger, Norway; 5 Department of Behavioral Sciences and Learning, Linköping University, Linköping, Sweden; 6 Centre for Ethics, Law and Mental Health (CELAM), University of Gothenburg, Gothenburg, Sweden; 7 Department of Psychology, University of Gothenburg, Gothenburg, Sweden; 8 Postgraduation Program in Public Health (PPGSC), University of Fortaleza (UNIFOR), Fortaleza, Brazil; 9 Institute of Mathematics and Computer Science, University of São Paulo, São Carlos, Brazil; 10 Nursing Department, Federal University of Ceará, Fortaleza, Brazil; 11 Center for Epidemiology and Community Medicine (CES), Region Stockholm, Stockholm, Sweden; 12 Center for Wellbeing, Welfare and Happiness, Stockholm School of Economics, Stockholm, Sweden

**Keywords:** Sarcopenia, Physical activity, Socioeconomic indicators, Older adults, Brazil

## Abstract

**Background:**

Sarcopenia is a progressive musculoskeletal disorder linked to physical disability, reduced quality of life, and increased mortality in older adults. While malnutrition is a well-established risk factor, understanding the behavioral (i.e., individual choices and habits) and social determinants of sarcopenia is crucial for designing effective public health strategies. This study investigates the associations between physical activity, sociodemographic and socioeconomic factors, and risk of sarcopenia in older adults living in Ceará, northeast Brazil.

**Methodology:**

This cross-sectional study included 736 older adults registered in primary health care units in the cities of Icó and Tauá, Ceará, from September 2022 to March 2023. Risk of sarcopenia was measured using the SARC-F questionnaire. Physical activity was assessed with the International Physical Activity Questionnaire for the Elderly (IPAQ-E), nutritional status with the Mini Nutritional Assessment, and sociodemographic and socioeconomic information through an adapted structured questionnaire. All instruments were administered in person by trained personnel. We analyzed associations using chi-square tests and logistic regression models.

**Results:**

Lower physical activity levels were strongly associated with greater odds of screening positive for risk of sarcopenia (*OR* = 7.99, *95% CI*: 5.00-12.77). We also found significant associations for sex, marital status, and occupation. Specifically, women, individuals who were widowed or divorced, and those without an occupation were more likely to screen positive for risk of sarcopenia. Nutritional status was, however, not significantly associated with risk for sarcopenia.

**Conclusions:**

Our findings demonstrate the importance of promoting physical activity and reducing social inequalities to prevent sarcopenia in aging populations. Public health strategies should be tailored to account for both behavioral and social determinants of health, with particular attention to the intersection of sex, social support, and occupation. Further longitudinal research is needed to clarify how these factors interact over time in the development of sarcopenia.

## Introduction

 Sarcopenia is a progressive and generalized skeletal muscle disorder [1, 2] characterized by a decline in muscle mass, strength, and function, particularly in older adults. It is associated with adverse outcomes including physical disability, falls, fractures, reduced quality of life, and increased mortality [3]. The World Health Organization (WHO) recognizes sarcopenia as a disease of the musculoskeletal system and connective tissue [4] that is not only about muscle mass loss, but rather a multifactorial condition involving complex interactions between biological, behavioral, and environmental factors [5]. This reflects an evolving understanding of sarcopenia as a dynamic syndrome shaped by multiple interrelated factors beyond aging itself. While sarcopenia is common among older adults, affecting an estimated 10 to 27% of this population worldwide [6], it may begin as early as the fourth decade of life in individuals exposed to early risk factors such as physical inactivity, malnutrition, or chronic disease [7–11]. Nonetheless, although malnutrition (e.g., inadequate protein intake) plays a well-established role in its development [11, 12], understanding the behavioral (i.e., individual choices such as physical activity habits) and social determinants of sarcopenia is crucial for developing effective public health strategies.

Physical inactivity is a key behavioral factor closely associated with the development and progression of sarcopenia [11, 12]. Sustaining regular physical activity, particularly through resistance training, has a crucial role in preserving muscle mass and strength across the lifespan [13, 14]. Evidence suggest that physically inactive older adults are significantly more likely to experience symptoms of sarcopenia than their more active counterparts [15]. This is because sedentary behavior contributes to musculoskeletal decline by reducing the mechanical and metabolic stimuli needed to maintain muscle mass, strength, and function [16, 17]. Such behavior also impairs muscle protein synthesis while increasing protein breakdown [18–21], accelerating the gradual loss of lean mass that accompanies natural aging [22]. In contrast, regular physical activity enhances functional capacity, and overall well-being [23–25], while also reducing the risk of chronic conditions such as cardiovascular disease, diabetes, and obesity—many of which are closely linked to the development of sarcopenia [26]. These findings underscore the importance of promoting active lifestyles as a core strategy for sarcopenia prevention and management.

While physiological mechanisms behind sarcopenia have been widely studied [27, 28], increasing evidence suggests that socioeconomic and sociodemographic factors also play a significant role in its development and progression [29]. For instance, individuals in socioeconomically vulnerable circumstances often face limited access to protein-rich, high-quality foods and safe spaces for regular physical activity [29–31]. Additionally, chronic diseases, such as diabetes and cardiovascular conditions, are disproportionally more prevalent in these populations, contributing further to muscle decline [32, 33]. Limited access to healthcare services, which is common in low-income populations, may also delay sarcopenia detection and complicate the treatment and management of the condition [29, 34].

Regarding sex-related differences in sarcopenia, women tend to report a higher prevalence of sarcopenia than men, a pattern influenced by both biological and social factors [35]. Although men generally maintain higher muscle mass through life, they experience rapid and pronounced muscle decline with age, particularly after the age of 60 [10], partially due to reductions in testosterone and growth hormone levels [36]. In contrast, women experience a gradual muscle loss, influenced by declining estrogen levels after menopause [37]. In other words, the effect of testosterone on muscle loss among men, might have a stronger effect than that of estrogen among women. However, differences in behavioral and structural factors also play a critical role. Older women are generally less likely than men to engage in strength training [38] and may face greater nutritional risk, caregiving burdens, and reduced access to health-promoting resources [10, 36].

### The present study

The multifactorial nature of sarcopenia present ongoing challenges for researchers and clinicians, highlighting the need to better understand its behavioral and social determinants across diverse populations [39]. As the global population ages, the public health implications of sarcopenia grow increasingly urgent, particularly among older adults living in socioeconomically vulnerable conditions. Yet, few studies have examined how physical activity, and socioeconomic and sociodemographic factors jointly relate to sarcopenia, especially in low- and middle-income settings. Expanding this evidence is essential for informing public health policies that reduce disparities and promote healthy aging. In this context, the present study investigates the associations between behavioral factors (i.e., physical activity), socioeconomic and sociodemographic factors (i.e., marital status, occupation, sex), and sarcopenia among older adults in the state of Ceará, in northeast Brazil.

## Methodology

### Ethical statement

This study complied with the ethical standards established by Resolution No. 466/12 of the Brazilian National Health Council [40], which regulates research involving human subjects. The study protocol was reviewed and approved by the Research Ethics Committee of the Federal University of Ceará via Plataforma Brasil [41] (approval No. 5.632.551). Before data collection, the managers of participating institutions signed the required consent and authorization forms. All participants received information about the purpose, procedures, potential risks, and ethical safeguards. Participation was anonymous and voluntary, and participants were informed of their right to withdraw at any time. Written informed consent was obtained from all individuals prior to participation.

### Participants and procedure

This cross-sectional observational study used primary data collected between September 2022 to March 2023 from older adults registered in primary health care units in the cities of Icó and Tauá, in the state of Ceará, in northeast Brazil. Data were obtained using validated instruments and an adapted socioeconomic questionnaire. Eligible participants were aged 60 years or older and registered in one of the selected primary health care units. Exclusion criteria included inability to communicate, a medical diagnosis of dementia (verified via medical records or caregiver reports), or cognitive impairment based on Mini-Mental State Examination scores below established cutoff points. Of the 816 individuals initially approached, 77 were excluded (69 for low Mini-Mental State Examination scores and 8 due to communication difficulties), resulting in a final sample of 739 individuals (444 females, 295 males) with an age mean of 70.43 (*SD* = 8.08).

Data collection was conducted in primary health care units that had an average of 80 patient visits per day, which was considered a significant number of appointments. The number of visits was verified through the coordinators of each unit. A combination of convenience sampling and active recruitment strategies was used, including approaching individuals in the primary health care facilities, during home visits, and at municipal health events and educational campaigns. Home visits were scheduled in advance with support from community health workers. Participants attending events were invited by community health workers to participate after activities concluded. This approach was chosen to facilitate access to a large number of older adults engaged with the public health system. All instruments were administered through structured face-to-face interviews conducted by trained personnel. Although data on comorbidities and medications were collected, these variables were not included in the present analysis.

## Measures

### Sarcopenia screening

Risk of sarcopenia was assessed using the SARC-F, a validated screening tool designed to identify individuals at increased likelihood of sarcopenia in clinical and research settings [42]^,^ [43–46]. The instrument includes five self-reported items covering strength (S), assistance with walking (A), rising from a chair (R), climbing stairs (C), and history of falls (F) [46]. Each item is scored on a scale from 0 to 2, yielding a total score ranging from 0 to 10. In this study, participants with a total score of ≥ 4 were classified as having risk of sarcopenia and those scoring 0 to 3 were classified as not at risk.

### Physical activity

Physical activity was assessed using the International Physical Activity Questionnaire for the Elderly (IPAQ-E), a validated instrument adapted from the original for use with adults aged 60 years and older [47, 48]. The instrument captures the frequency (i.e., number of days during the last week) and duration (i.e., minutes per day) of vigorous, moderate, and walking/sitting activities across multiple physical activity domains including leisure time, domestic, occupational, and transport-related activities, as well as sedentary behavior. Weekly energy expenditure was estimated in metabolic equivalence (MET) minutes, which is calculated by multiplying frequency by duration by the corresponding MET value for each activity (i.e., 3.3 for walking, 4.0 for moderate activity, and 8.0 for vigorous activity) [49]. The MET-minutes from all activity types were then summed up to obtain the total weekly energy expenditure. Based on standard IPAQ scoring procedures, participants were classified into two categories: less active (< 600 MET-min/week) and more active (≥ 600 MET-min/week).

### Nutritional status

Nutritional status was measured using the Mini Nutritional Assessment, a validated 18-item tool widely used in older adults [50]. It identifies individuals at risk of malnutrition or malnourished by evaluating factors such as dietary intake, recent weight loss, mobility, psychological stress, and anthropometrics (e.g., BMI and calf circumference). Each item is individually scored, with a total score ranging from 0 to 30 points. Based on established cut-offs points, participants were classified into three categories: normal nutritional status (≥ 24.0 points), at risk of malnutrition (17.0–23.5 points), and malnourished (< 17.0 points). In our analysis, participants classified as at risk of malnutrition and malnourished were combined into a single group. This decision was based on the relatively low prevalence of malnourished individuals in the sample and the shared clinical significance of both categories as indicators of a compromised nutritional status. Grouping these categories allowed for more robust comparisons while preserving the distinction between individuals with adequate nutritional status and those exhibiting any degree of nutritional vulnerability.

### Socioeconomic and sociodemographic indicators

Socioeconomic data was obtained with the use of an adapted questionnaire [51]. The following variables were used in the present study: marital status (single, married or cohabiting, widowed or divorced), current occupation (no occupation, farming, commerce/other occupations), education (illiterate, 1–4 years, 5–8 years, 9–11 years, and more than 12 years), monthly income (categorized by the amount of national minimum wage salaries per month for a household), ethnicity (self-reported), health care unit location (urban or rural, based on primary health care unit registry), sex (male/female), age (collected in total years and categorized into 60 to 70 years, 71 to 80 years, and over 80 years). The categorization of occupation, based on the prominence of subsistence and informal labor in Brazil, and the categorization of income reflect patterns commonly used in Brazilian public health research. While this categorization allows for clearer comparisons and statistical power, there is potential for residual confounding and misclassification due to its simplified nature, which may not capture the full socioeconomic heterogeneity of the population.

### Statistical analysis

Following data validation, three cases were excluded due to input errors, yielding a final sample of 736 participants (60.05% women, 39.95% males). Descriptive statistics were computed for all variables. Bivariate associations between sarcopenia classifications, based on SARC-F scores, and categorical predictors were examined using chi-square tests of independence. To assess the influence of predictor variables on sarcopenia, we constructed binary logistic regression models, since the dependent variable was dichotomous (i.e., no risk vs. probable sarcopenia). Regression coefficients were expressed as odds ratios (ORs) with 95% confidence intervals (CIs), representing the exponential increase in the likelihood of risk of sarcopenia. Four logistic regression models were specified:

- Model 0 (Null model) established a baseline probability of probable sarcopenia without the influence of any predictor variables and served as a reference point for evaluating whether subsequent models, including behavioral and social factors, provide improved predictive value;
- Model 1 included behavioral (physical activity) and nutritional status;
- Model 2 added socioeconomic and sociodemographic variables (e.g., marital status, occupation);
- Model 3 was generated via backward stepwise logistic regression using the Akaike Information Criterion (AIC) to identify the most parsimonious model.

Chi-square tests of independence were performed using the SciPy library in Python. Logistic regression models were estimated using the glm function in the stats package in R, and the step AIC function from the MASS package for model selection. In this study, a p-value of < 0.05 was considered statistically significant.

## Results

### Descriptive characteristics

The final sample included 736 older adults, of whom 60.05% were women and 39.95% were men. Most participants were self-identified as brown or black (66.71%), retired (96.60%), and illiterate (46.74%). Based on SARC-F scores, 25.41% (*n* = 187) were classified at risk of sarcopenia (SARC-F ≥ 4). In terms of physical activity, 52.72% of participants were classified as less active (< 600 MET-min/week). Table 1 summarizes the descriptive statistics.

**Table 1:** Descriptive statistics for all study variables, presented as counts and percentages

Variables	Category	Frequency	Percentage
Sarcopenia	No risk of sarcopenia	549	74.59
	Risk of sarcopenia	187	25.41
Physical activity	More active	348	47.28
	Less active	388	52.72
Primary Health Care Unit	Rural area	359	48.78
	Urban area	377	51.22
Age	60 to 70 yrs.	418	56.79
	71 to 80 yrs.	223	30.30
	> 80 yrs.	95	12.91
Sex	Female	442	60.05
	Male	294	39.95
Education	Illiterate	344	46.74
	1 to 4 yrs.	243	33.01
	5 to 8 yrs.	82	11.14
	9 to 11 yrs.	18	2.45
	> 12 yrs.	49	6.66
Income	< 1 National Minimum Wage	7	0.96
	1 to 2 National Minimum Wage	643	87.36
	> 3 National Minimum Wage	86	11.68
Occupation	No occupation	553	75.14
	Farming	152	20.65
	Commerce/other occupations	31	4.21
Retired	Yes	711	96.60
	No	25	3.40
Marital status	Single	61	8.29
	Married or cohabiting	464	63.04
	Widowed or divorced	211	28.67
Self-reported ethnicity	White	245	33.29
	Brown or Black	491	66.71
Nutritional status	Normal nutritional status	683	92.80
	At risk of malnutrition	53	7.20

Note. Sarcopenia classification was based on SARC-F score: ≥ 4 = risk of sarcopenia; 0–3 = no risk of sarcopenia. Physical activity classification was based on scores in the IPAQ-E (MET-minutes/week): less active < 600 MET-min/week and more active ≥ 600 MET-min/week. Nutritional status was based on Mini Nutritional Assessment scores: normal nutritional status ≥ 24.0 points, at risk of malnutrition = 17.0–23.5.0.5 points, and malnourished < 17.0 points

### Bivariate associations between categorical variables and SARC-F condition

Chi-square tests revealed significant associations between the risk of sarcopenia classification and several variables. Physical inactivity was strongly associated with sarcopenia classification (*x²* = 113.86, *p* <.0001). Sociodemographic and socioeconomic variables, including occupation (*x²* = 40.60, *p* <.0001), marital status (*x²* = 19.22, *p* <.0001), sex (*x²* = 13.43, *p* <.05), age (*x²* = 10.58, *p* <.05), retired (*x²* = 5.14, *p* <.05), and nutritional status (*x²* = 3.91, *p* <.05) were also significantly associated. Income (*x²* = 3.01, *p* =.22), primary care unit (*x²* = 0.64, *p* =.42), ethnicity (*x²* = 0.34, *p* =.56), and education (*x²* = 1.47, *p* =.83) were not significantly associated with sarcopenia classification.

### Multivariate logistic regression models

The null model served as a baseline, and it included no predictor variables. It estimated only the intercept, which had a value of −1.08 (*SE* = 0.08, *p* <.001, *OR* = 0.34), corresponding to a constant predicted probability of 0.25 for risk of sarcopenia across the sample (see Table 2). Using the classification threshold of 0.50, the null model predicts that no individuals would be classified as risk of sarcopenia. The model’s Akaike Information Criterion (AIC) was 836.28, indicating relatively poor model fit compared to the subsequent models that included predictors.

Model 1 included physical activity level and nutritional status as predictor of sarcopenia classification. The inclusion of these variables substantiable improved model fit, as indicated by a reduction in AIC from 836.28 (null model) to 712.82. Among the predictors, physical activity was a statistically significant predictor. Participants classified as less active (i.e., < 600 MET-min/week) had significantly greater odds of screening for risk of sarcopenia compared to more active individuals (*OR* = 9.14, *p* <.001). Nutritional status, on the other hand, was not a significant predictor in this model (*OR* = 1.18, *p* =.59). These results indicate that older adults who were physically inactive were more than nine times more likely to exhibit risk of sarcopenia compared to their more active counterparts, even after accounting for nutritional status (see Table 2).

Model 2 was developed to assess the joint effects of physical activity, nutritional status, and a range of sociodemographic and socioeconomic variables on the likelihood of risk of sarcopenia. As shown in Table 2, this model yielded a lower AIC value (698.30), indicating improved model fit compared to previous models. Physical activity remained a robust and statistically significant predictor of sarcopenia (*OR* = 8.30, *p* <.001), with less active individuals having over eight times greater odds for risk of sarcopenia compared to their more active counterparts. Nutritional status, on the other hand, was not significantly associated with sarcopenia in this model (*OR* = 1.03, *p* =.93), consistent with findings in Model 1. Among the sociodemographic and socioeconomic variables, sex (*OR* = 0.58, *p* =.01), occupation (*OR* = 0.38, *p* =.01) and marital status (*OR* = 1.61, *p* =.03) were statistically significant predictors of sarcopenia: female participants had 58% greater odds of screening for risk of sarcopenia compared to males, individuals engaged in farming had 62% lower odds of screening for risk of sarcopenia compared to those with no occupation, and being widowed or divorced was associated with 61% greater odds of screening for risk of sarcopenia compared to those married or in a stable union. Besides sex and occupation, educational attainment also showed a marginally significant association (*OR* = 0.63, *p* =.05). Specifically, individuals with 1 to 4 years of formal education had 37% lower odds of screening for risk of sarcopenia compared to those with no formal education, suggesting a potential protective effect of even minimal schooling. See details in Table 2.

Model 3 was developed using a stepwise logistic regression approach with AIC as the model selection criterion. As shown in Table 2, this model yielded the lowest AIC value (687.54) among all models tested, indicating the best overall fit to the data. In this model, physical activity remained a robust and statistically significant predictor of screening for risk of sarcopenia, with less active individuals showing nearly eight times greater odds of screening positive for risk of sarcopenia compared to more active individuals (*OR* = 7.99, *p* <.001). Sex also remained a significant factor, with female participants having approximately 62% greater odds of risk of sarcopenia compared to males (*OR* = 0.62, *p* =.02), consistent with previous models. Occupation showed a significant association as well. Participants engaged in farming had 63% lower odds of screening for risk of sarcopenia compared to those without an occupation (*OR* = 0.37, *p* =.01), suggesting a potential protective effect of physically active work. Finally, marital status remained a relevant predictor. Being widowed or divorced was associated with 58% greater odds of risk of sarcopenia compared to being married or in a stable union (*OR* = 1.58, *p* =.03). Other marital categories (e.g., single) did not reach statistical significance.

**Table 2:** Summary of logistic regression models assessing predictors of risk of sarcopenia

Model	Coefficient	Reference Category	AIC	Estimate (β, log-odds)	Standard error	*p*-value	95% confidence interval (log-odds)		OR	95% confidence interval (OR)	
Null Model	(Intercept)		836.28	−1.08	0.08	0.001	−1.24	−0.91	0.34	0.29	0.40
Model 1	(Intercept)		712.82	−2.56	0.21	0.001	−2.97	−2.16	0.08	0.05	0.12
	Physical Activity	Less active		2.21	0.23	0.001	1.76	2.67	9.14	5.79	14.43
	Nutritional status	At risk of malnutrition		0.17	0.32	0.59	−0.45	0.79	1.18	0.64	2.19
Model 2	(Intercept)		698.30	−0.28	1.53	−0.181	−3.28	2.73	0.76	0.04	15.27
	Physical activity	Less active		2.12	0.25	0.001	1.63	2.60	8.30	5.11	13.48
	Nutritional status	At risk of malnutrition		0.03	0.34	0.93	−0.64	0.70	1.03	0.53	2.01
	Primary Care Unit	Urban area		0.08	0.20	0.68	−0.31	0.47	1.08	0.74	1.60
	Sex	Male		−0.54	0.22	0.01	−0.97	−0.11	0.58	0.38	0.90
	Age	71 to 80 years		−0.31	0.23	0.18	−0.77	0.14	0.73	0.46	1.15
		> 80 years		0.09	0.30	0.75	−0.49	0.67	1.10	0.61	1.96
	Education	1 to 4 years		−0.46	0.23	0.05	−0.92	0	0.63	0.40	1.00
		5 to 8 years		−0.61	0.33	0.07	−1.27	0.04	0.54	0.28	1.04
		9 to 11 years		−0.7	0.75	0.35	−2.16	0.77	0.50	0.11	2.17
		> 12 years		0.01	0.43	0.98	−0.84	0.86	1.01	0.43	2.36
	Income	1 to 2 NMW		−1.71	1.51	0.26	−4.67	1.24	0.18	0.01	3.46
		> 3 NMW		−1.39	1.52	0.36	−4.37	1.60	0.25	0.01	4.97
	Occupation	Farming		−0.97	0.38	0.01	−1.71	−0.23	0.38	0.18	0.79
		Commerce/other occupations		−0.86	0.68	0.21	−2.21	0.48	0.42	0.11	1.61
	Retired	No		−1.83	1.39	0.19	−4.56	0.89	0.16	0.01	2.44
	Marital status	Widowed or divorced		0.48	0.21	0.03	0.06	0.89	1.61	1.06	2.44
		Single		0.36	0.36	0.31	−0.34	1.07	1.44	0.71	2.91
	Self-reported ethnicity	Brown or Black		−0.13	0.21	0.52	−0.54	0.27	0.88	0.58	1.31
Model 3	(Intercept)		687.54	−2.3	0.23	0.001	−2.75	−1.84	0.10	0.06	0.16
	Physical activity	Less active		2.08	0.24	0.001	1.61	2.55	7.99	5.00	12.77
	Sex	Male		−0.48	0.21	0.02	−0.9	−0.07	0.62	0.41	0.94
	Occupation	Farming		−0.99	0.36	0.01	−1.7	−0.29	0.37	0.18	0.75
		Commerce/other occupations		−1.17	0.64	0.07	−2.43	0.09	0.31	0.09	1.10
	Marital status	Widowed or divorced		0.46	0.21	0.03	0.05	0.86	1.58	1.06	2.36
		Single		0.33	0.35	0.34	−0.35	1.01	1.39	0.71	2.75

Note. Odds ratios (OR) reflect the odds of risk of sarcopenia relative to these reference groups. Sarcopenia classification was based on SARC-F score: ≥ 4 = risk of sarcopenia; 0–3 = no risk of sarcopenia. Physical activity classification was based on scores in the IPAQ-E (MET-minutes/week): less active < 600 MET-min/week and more active ≥ 600 MET-min/week. Nutritional status was based on Mini Nutritional Assessment scores: normal nutritional status ≥ 24.0 points, at risk of malnutrition = 17.0–23.5.0.5 points, and malnourished < 17.0 points

## Discussion

The present study aimed to explore the associations between physical activity, socioeconomic indicators, and sarcopenia in a population of older adults in the state of Ceará, Brazil. The findings emphasize the multifactorial nature of sarcopenia, and the importance of both lifestyle and socioeconomic indicators in the development and progression of the condition. Our results have shown a particularly strong association between physical activity levels and risk of sarcopenia, which are consistent with numerous studies demonstrating the protective effects of physical activity in preventing the development of the condition [22, 38, 52–54]. Previous research shows that physically inactive older adults have a significantly higher risk of developing sarcopenia, as a sedentary lifestyle reduces muscle growth stimuli and accelerates muscle loss [16].

The association between higher levels of physical activity and a reduced risk of sarcopenia is due to the positive and direct impact of physical activity on muscle mass and strength [53]. Older individuals who are physically active exhibit higher levels of cardiorespiratory fitness, upper and lower body strength, and tend to avoid sedentary behavior, which significantly contributes to a reduced risk of developing the condition [55]. Regular physical activity, in particular resistance training, has been shown to enhance muscle protein synthesis, improve muscle mass and strength, and mitigate the age-related decline in muscle function [56, 57]. Also, the mechanical stresses caused by resistance training on skeletal muscle, stimulate specialized satellite cells responsible for muscle repair and growth [58], helping to counteract the natural muscle degradation associated with aging. Furthermore, physical activity enhances the efficiency of neuromuscular junctions, improving the communication between nerve and muscle cells [59]. It also increases circulatory flow, ensuring better oxygenation and nutrient delivery, both of which are essential for muscle maintenance and repair [60]. Regular physical activity reduces systemic inflammation and oxidative stress; which are major contributors to muscle degeneration in aging populations [61]. It also promotes the release of anabolic hormones, such as testosterone and growth hormone, further supporting muscle preservation and function in older adults [56].

Nonetheless, along muscle-strengthening exercises, WHO recommends older individuals to engage in at least 150 min of moderate-intensity aerobic activity per week on two or more days. Specifically, older adults should also incorporate multicomponent physical activities, such as balance and strength training, at a moderate or higher intensity on at least three days per week in order to improve functional capacity and reduce the risk of falls [26]. In brief, physical activity is highly effective for the prevention and management of the condition, with research indicating that even smaller amounts of physical activity can significantly reduce the risk of sarcopenia [38, 57], provide substantial health benefits and improve the functional capacity of older adults [22].

Similarly to physical activity levels, several sociodemographic and socioeconomic indicators, particularly sex, marital status, and occupation, were significantly associated with the risk of sarcopenia. As noted in the Introduction, although men generally maintain greater muscle mass throughout life, they tend to experience a sharper and more rapid decline in muscle mass and strength after age 60 due to hormonal shifts, such as reduced testosterone and growth hormone levels [62, 63]. In contrast, women experience a more gradual muscle loss, largely driven by the post-menopausal drop in estrogen, which contributes to reduced muscle protein synthesis and increased muscle degradation [64]. These hormonal patterns help explain some of the sex differences observed in our findings, where male participants had a significantly lower risk of sarcopenia than females. Our results also reinforce the role of behavioral and structural factors in shaping these outcomes. As discussed previously, older women are often less likely to engage in resistance training, and may face compounded vulnerabilities due to nutritional risk, caregiving responsibilities, and unequal access to health-promoting resources. This multifaceted interplay between biological, behavioral, and social factors likely contributes to the higher prevalence of sarcopenia symptoms observed among women in our study.

Building on the observed sex differences, nutritional vulnerability may further add to older women’s risk of sarcopenia. Inadequate intake of protein, vitamins, and minerals impairs muscle repair and regeneration [10], and women in lower socioeconomic groups are particularly affected due to reduced access to high quality nutrition [2]. These nutritional challenges are often exacerbated by lower income, food insecurity, and limited access to healthcare services. In addition, older women are generally less likely to engage in resistance training [38], a key intervention for preserving muscle mass and strength, due to social, cultural, and structural barriers (e.g., limited access to healthcare). Together, these intersecting disadvantages may increase the risk of sarcopenia among older women by both biological and behavioral pathways [22]. Despite this, our study did not find a statistically significant association between nutritional status and sarcopenia risk. This lack of effect may be explained, in part, by the relatively low proportion of participants classified as at risk of malnutrition (7.20%), which may have limited statistical power to detect significant associations. In addition, the instrument used to assess nutritional status combines objective and self-reported elements and may be subject to recall or social desirability bias. It is also possible that the effect of nutritional status on risk of sarcopenia overlaps with, or is mediated by, other variables included in the model, such as physical activity and socioeconomic factors. Future studies with more detailed dietary assessments and larger, more diverse samples may help clarify the nature of this relationship.

We also found that widowed or divorced individuals had a significantly higher likelihood of risk of sarcopenia compared to those who were married or cohabiting, suggesting a strong link between social support and muscle health in older adults. In the stepwise model, being widowed or divorced was associated with a 58% higher likelihood of exhibiting risk of sarcopenia, even after adjusting for other variables. This finding reinforces the importance of social relationships in mitigating the risk of sarcopenia. The loss of a partner through widowhood, divorce, or separation often increases the risk of social isolation and living alone, both of which are associated with poor health behaviors, reduced physical activity, and diminished nutritional intake later in life [65, 66]. These underlines the importance of early screening and the design of person-centered, community-based interventions targeting these socially isolated older adults [30, 65, 66].

Occupation status also merged as a significant predictor of risk of sarcopenia. Specifically, individuals engaged in farming had a substantially lower likelihood of risk of sarcopenia, approximately 62% less, compared to those without an occupation. This supports existing evidence that physically demanding work can serve as a protective factor for musculoskeletal health by preserving muscle mass, maintaining strength, and attenuating age-related muscle decline throughout the life course [22, 67]. Similarly to resistance training, regular engagement in manual labor stimulates muscle protein synthesis, enhances neuromuscular function, and reduces the risk of muscle atrophy. In contrast, those without an occupation, often leading more sedentary lifestyles, may be at greater risk due to the lack of regular physical activity stimulation required to maintain muscle function [56, 57, 68].

Although the recognition of sarcopenia as a public health concern is relatively recent, its consequences, particularly in vulnerable populations, warrant urgent attention [28]. Sarcopenia is strongly associated with disability, falls, hospitalization, and mortality, reinforcing the need for preventive strategies that include early screening, timely diagnosis, and effective treatment [69]. In this context, the implementation of public health policies that promote physical activity, provide nutritional support, and improve healthcare accessibility can significantly reduce the prevalence of sarcopenia and improve the quality of life of vulnerable older adults [9, 13, 29]. Indeed, the present findings have practical implications for primary health care, particularly in low-resource settings such as Brazil’s northeast. For example, incorporating simple and cost-effective screening tools like the SARC-F into routine assessments can facilitate early detection of individuals at risk of sarcopenia. Additionally, multidisciplinary approaches that promote physical activity, provide nutritional support, and address social vulnerability are critical for reducing disparities in sarcopenia outcomes. Public health initiatives aimed at increasing access to exercise programs, improving dietary resources, and strengthening social support structures may significantly improve quality of life and reduce functional decline in aging populations.

### Strengths and limitations

Our study offers several notable strengths in advancing the understanding of sarcopenia and its associated risk factors in older populations. Foremost, it provides a comprehensive investigation of both behavioral (i.e., physical activity) and both sociodemographic and socioeconomic determinants in a population of older adults in northeastern Brazilian. The use of well-established and validated assessment tools, such as the SARC-F questionnaire for risk of sarcopenia screening and the IPAQ-E for physical activity assessment, adds to the methodological rigor of the study. Another strength lies in the relatively large sample size (*n* = 736), which ensured sufficient statistical power and improves the generalizability of the findings within the study population. Additionally, the focus on older adults in a socioeconomically vulnerable region (Ceará, Brazil) enhances the relevance of the research by shedding light on a group that may face unique challenges related to aging and functional decline.

Despite its contributions, the study also has limitations that should be acknowledged. The cross-sectional design restricts the ability to draw causal inferences. While significant associations between physical activity, socioeconomic indicators, and risk of sarcopenia were identified, the temporal direction of these relationships remains unclear. Future research using longitudinal designs will be essential to better understand how these factors interact over time and establish causal pathways for the development and progression of sarcopenia over time. Another limitation concerns the sampling strategy. Participants were selected from public primary health care units using a convenience sampling approach, which may introduce selection bias. Older adults not engaged with the public health system, such as those receiving private care or with limited access to health services, were likely underrepresented. Furthermore, individuals who participate in community outreach or health programs may systematically differ from those who do not, potentially limiting the generalizability of results [70]. The exclusion of individuals with severe cognitive impairments or communication difficulties may also have led to an underrepresentation of a particularly vulnerable subgroup.

The study’s reliance on self-reported measures to assess physical activity, nutritional status, and risk of sarcopenia, might be subject to recall and social desirability bias. For instance, although the SARC-F is a validated tool [8] and practical for use in community settings [9, 10], future studies would benefit from the inclusion of objective measures, such as accelerometers for physical activity, handgrip strength dynamometry, gait speed tests, and imaging-based techniques like Dual-Energy X-ray Absorptiometry or Bioelectrical Impedance Analysis to assess body composition and muscle mass [5–7]. While these methods may be less feasible in resource-limited contexts, they could improve the precision and robustness of screening for and research on sarcopenia. Additionally, the potential for residual confounding cannot be ruled out. Important health-related factors such as comorbidities, medication use, inflammatory conditions, or hormonal levels were not captured in the analyses and may have influenced the observed associations [71, 72]. Finally, findings should be interpreted with caution given the specific geographic and cultural setting. The socioeconomic and lifestyle characteristics of older adults in Icó and Tauá may differ considerably from other Brazilian regions or international contexts, which could limit the broader applicability of the findings.

## Conclusions

This study emphasizes the complex interactions between physical activity, sociodemographic and socioeconomic indicators, and the risk of sarcopenia among older adults in Brazil. The findings suggest that there is a need for person-centered public health strategies that not only promote physical activity but also address structural disparities that shape health outcomes in aging populations. Effectively prevention and management of sarcopenia require a multidimensional approach that considers the behavioral, nutritional, and both sociodemographic and socioeconomic determinants. Promoting physical activity and improving access to high-quality nutrition should be prioritized in public health policies, particularly in low-resource settings. Equally important is the need to reduce structural barriers related to income, education, occupation, and social support—factors that disproportionately affect vulnerable groups. By tailoring interventions to the specific needs of diverse groups, health systems can reduce disparities in sarcopenia outcomes and support healthier aging. Simple tools such as the SARC-F can aid in early detection, while community-based and person-centered strategies[73–76] that integrate social, nutritional, and physical health support can foster resilience among older adults. Further research, particularly longitudinal designs and objective measures, is needed to better understand causal mechanisms and refine interventions. Ultimately, targeting both the behavioral and social determinants of sarcopenia will be key to advancing health equity and improving quality of life in aging populations.

*''A society that does not value its older people denies its roots and endangers its future.''*

*— Nelson Mandela*.

## Data Availability

The data supporting the findings of this study are available from the research group, but restrictions apply to the availability of these data, and the data are not publicly available. In case data is requested, please contact Matheus Guerra.
